# Intrinsic valley Hall transport in atomically thin MoS_2_

**DOI:** 10.1038/s41467-019-08629-9

**Published:** 2019-02-05

**Authors:** Zefei Wu, Benjamin T. Zhou, Xiangbin Cai, Patrick Cheung, Gui-Bin Liu, Meizhen Huang, Jiangxiazi Lin, Tianyi Han, Liheng An, Yuanwei Wang, Shuigang Xu, Gen Long, Chun Cheng, Kam Tuen Law, Fan Zhang, Ning Wang

**Affiliations:** 1 Department of Physics and the Center for Quantum Materials, the Hong Kong University of Science and Technology, Hong Kong, China; 2 Department of Physics, University of Texas at Dallas, Richardson, TX 75080 USA; 3 School of Physics, Beijing Institute of Technology, 100081 Beijing, China; 4 Department of Materials Science and Engineering, Southern University of Science and Technology, 518055 Shenzhen, China

## Abstract

Electrons hopping in two-dimensional honeycomb lattices possess a valley degree of freedom in addition to charge and spin. In the absence of inversion symmetry, these systems were predicted to exhibit opposite Hall effects for electrons from different valleys. Such valley Hall effects have been achieved only by extrinsic means, such as substrate coupling, dual gating, and light illuminating. Here we report the first observation of intrinsic valley Hall transport without any extrinsic symmetry breaking in the non-centrosymmetric monolayer and trilayer MoS_2_, evidenced by considerable nonlocal resistance that scales cubically with local resistance. Such a hallmark survives even at room temperature with a valley diffusion length at micron scale. By contrast, no valley Hall signal is observed in the centrosymmetric bilayer MoS_2_. Our work elucidates the topological origin of valley Hall effects and marks a significant step towards the purely electrical control of valley degree of freedom in topological valleytronics.

## Abstract (web summary)

Electrons hopping in two-dimensional honeycomb lattices possess a valley degree of freedom. Here, the authors observe room-temperature valley Hall transport without any extrinsic symmetry breaking in the non-centrosymmetric monolayer and trilayer MoS_2_ by purely electronic means, whereas no valley signal is detected for centrosymmetric bilayer MoS_2_.

## Introduction

Electron valley degree of freedom emerges as local extrema in the electronic band structures. Inequivalent valleys, well separated in the Brillouin zone, can be energetically degenerate due to symmetry and serve as novel information carriers controllable via external fields^1–6^. A feasible means to manipulate such a valley degree of freedom is through a valley Hall effect (VHE)^5–9^. Analogous to an ordinary Hall effect, in which a transverse charge current is driven by a uniform magnetic field in real space, a transverse valley current in the VHE is produced by valley-contrasting Berry curvatures in momentum space. Upon the application of an external electric field, the curvatures drive carriers from different valleys to traverse in opposite directions. Therefore, the VHE has been a major theme in the study of valleytronics, particularly in those 2D materials featuring K and K’ valleys in their hexagonal Brillouin zones^10–19^.

**Fig. 1:**
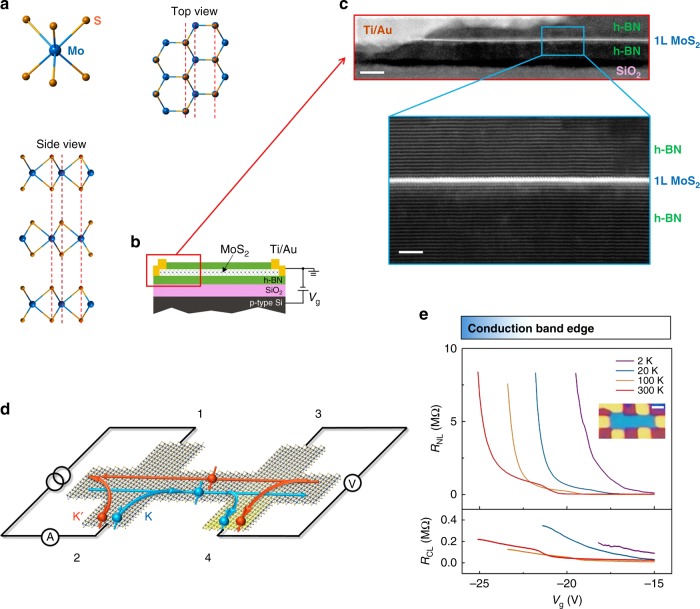
Valley Hall transport induced nonlocal resistance in monolayer MoS_2_. **a** Top view and side view of the crystal structure of 2H-MoS_2_; an odd- (even-) layer is inversion asymmetric (symmetric). **b** Schematic of the h-BN encapsulated MoS_2_ field-effect transistor. **c** High-resolution bright-field STEM image showing details of the edge-contacted monolayer MoS_2_ device structure (scale bar 10 nm). The expanded region shows that the BN-MoS_2_-BN interface is pristine and free of impurities down to the atomic scale (scale bar 3 nm). **d** Schematic of the nonlocal resistance measurement and the VHE-mediated nonlocal transport. The applied charge current in the left circuit generates a pure valley current in the transverse direction via a VHE. This valley current induces opposite chemical potential gradients for the two valleys over the inter-valley scattering length, which, in turn, generates a voltage drop measured by probes 3 and 4 in the right circuit via an inverse VHE. **e** Nonlocal resistance *R*_NL_ (upper panel) and the classical ohmic contribution *R*_CL_ (lower panel) as functions of gate voltage *V*_g_ at varied temperatures. Inset: optical image of a typical monolayer MoS_2_ device (scale bar 2 μm). A MoS_2_ Hall bar is sandwiched between the top and bottom h-BN flakes

As Berry curvature is even under spatial inversion (*P*) and odd under time reversal (*T*), the VHE cannot survive when both *P* and *T* symmetries are present. To achieve VHEs in monolayer and bilayer graphene, an elaborately aligned h-BN substrate^10^ and a strong dual gating field^11,12^ were respectively utilized to break the *P* symmetry. To excite VHEs in specific valleys^17,18^, circularly polarized lights^20–22^ were used for breaking the *T* symmetry in atomically thin transition-metal dichalcogenides (TMDC). Monolayer TMDCs have direct band gaps of optical frequencies at two inequivalent K-valleys^23,24^, due to the intrinsic *P* asymmetry in their unit cells depicted in Fig. 1a. Thus, Berry curvatures with opposite signs naturally emerge at the two K-valleys. Moreover, the *T* and mirror symmetries lock the spin and valley indices of the sub-bands split by the spin-orbit couplings, both of which are flipped under *T*; the spin conservation suppresses the inter-valley scattering. Therefore, monolayer TMDCs have been deemed an ideal platform for realizing intrinsic VHE without extrinsic symmetry breaking^15,16^.

However, the quantum transport in atomically thin TMDCs has been a long-standing challenge due to the low carrier mobility and the large contact resistance in their field-effect devices prepared by an exfoliation method. Recent breakthroughs in the fabrication of low-temperature ohmic contacts for high-mobility 2D TMDC devices^25–28^ have already facilitated the observation of transport hallmarks of Q-valley electrons^28,29^, K-valley electrons^30,31^, K-valley holes^32–34^, and Γ-valley holes^35^. These discoveries have revealed the rich and unique valley physics in the platform of atomically thin TMDCs.

In this work, we design nonlocal, layer-dependent, transport measurements to systematically examine the intrinsic VHEs in n-type 2H-MoS_2_. For the first time, we observe nonlocal resistances that exhibit cubic power-law scaling with the local resistances in the monolayers and trilayers, evidencing intrinsic VHEs. Because of the large intrinsic bandgaps and spin-valley locking of TMDCs, such VHEs can even be observed at room temperature in our monolayer devices. Beyond critical carrier densities (∼4.0 × 10^11^ cm^−2^ for monolayers and trilayers), the cubic scaling turns into linear scaling. Notably, only linear scaling is observed in bilayer MoS_2_, where the *P* symmetry is restored. Intriguingly, although the monolayer and trilayer feature respectively K- and Q-valleys near their conduction-band edges, they display comparable valence-band Berry curvatures, valley Hall signatures, and micron-sized valley diffusion lengths. Our results not only offer the first experimental evidence for the intrinsic VHE but also help elucidate its topological origin^6^ in odd-layer TMDCs and pave the way for realizing room-temperature low-dissipation valleytronics by purely electronic means.

## Results

### Devices for nonlocal measurements

The structure of a monolayer MoS_2_ field-effect transistor is sketched in Fig. 1b. Its bright-field cross-sectional scanning transmission electron microscopy (STEM) image in Fig. 1c clearly shows the layered BN-MoS_2_-BN structure without any impurities in the interfaces down to the atomic scale. The device fabrication process includes a dry transfer step followed by a reactive ion etching step^27,28,35^ (see Methods and Supplementary Fig. 1 for details). A low contact barrier formed on the *n*-type MoS_2_ is evidenced by the *I*-*V* curves, contact resistances (Supplementary Fig. 2), and the field-effect mobilities *μ* varied from 500–4000 cm^2^ V^−1^ s^−1^ for monolayers, 4000–23000 cm^2^ V^−1^ s^−1^ for bilayers, and 10000–25000 cm^2^ V^−1^ s^−1^ for trilayers at *T* = 2 K (Supplementary Figs. 3 and 4). The impurity-free STEM images and the high mobilities coincide well with the low residue carrier densities (*n*^∗^ = 4 × 10^10^ cm^−2^, see Supplementary Fig. 5).

As for the electronic measurement, an inverse VHE is exploited to detect a valley current, as sketched in Fig. 1d. An applied current *I*_12_ through probes 1 and 2 induces charge imbalance in a remote region, as measured by the voltage drop *V*_34_ between probes 3 and 4 (Supplementary Fig. 6). The nonlocal resistance *R*_NL_ = *V*_34_/*I*_12_ mediated by the valley Hall current was predicted^36^ to present cubic power-law dependence on the local resistance *R*_L_ = *V*_24_/*I*_13_.

### Nonlocal transport in monolayer MoS_2_

Nonlocal resistance *R*_NL_ in an n-type monolayer MoS_2_ (sample B of length *L* = 6 μm and width *W* = 1.5 μm illustrated in the inset of Fig. 1e), measured as a function of gate voltage *V*_g_ at varied temperatures, is shown in Fig. 1e. A giant *R*_NL_ is observed in the range of *V*_g_ ∼ −15 to −25 V that amounts to the electron density *n* ∼ 10^10^ to 10^11^ cm^−2^. In particular, the observed *R*_NL_ ∼ 10^6^ Ω exceeds the classical ohmic contribution $R_{{\mathrm{CL}}} = R_{\mathrm{L}}\frac{W}{{{\mathrm{\pi }}L}}{\mathrm{e}}^{ - {\mathrm{\pi }}L/W} \sim 10^4\,\Omega$ by two orders of magnitude in the range of *V*_g_ ∼ −15 to −18 V at 2 K and *V*_g_ ∼ −22 to −25 V at 300 K. Another unexpected feature of *R*_NL_ is its *V*_g_ dependence. In sharp contrast to the classical contribution *R*_CL_, which decreases gradually with increasing *V*_g_, the observed *R*_NL_ drops by at least one order of magnitude within an increase of several volts in *V*_g_. Both the pronounced nonlocal signal and its unusual sensitivity to *V*_g_ suggest that the observed *R*_NL_ has a physical origin different from the classical ohmic contribution *R*_CL_.

**Fig. 2:**
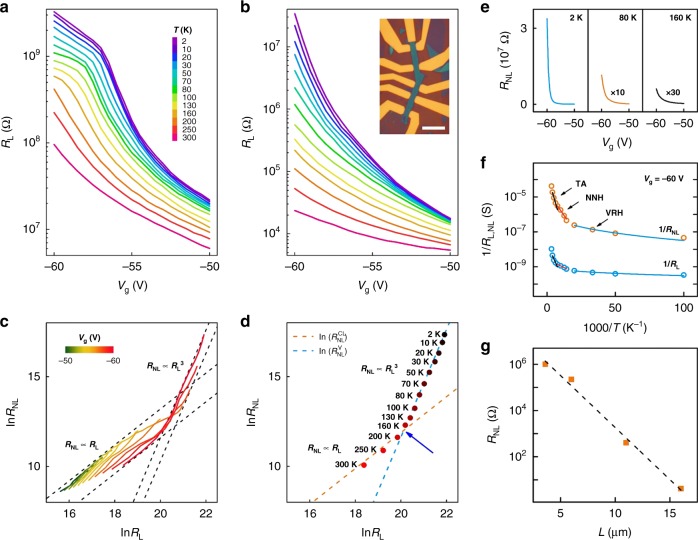
Local and nonlocal resistances of monolayer MoS_2_. **a**, **b** Semilog plots of *R*_L_ and *R*_NL_ as a function of *V*_g_ measured at varied temperatures. Inset of **b**: optical micrograph of our typical h-BN/MoS_2_/h-BN device with multi-terminal Hall Bar configurations. Scale bar: 5 μm. **c** Scaling relations between ln *R*_L_ and ln *R*_NL_ at *V*_g_ ranging from −50 V to −60 V. When the electron density is relatively high, i.e., *R*_L_ and *R*_NL_ are small, *R*_NL_ is linearly proportional to *R*_L_. When the electron density is relatively low, a crossover from linear to cubic scaling is observed. The critical density *n*c = 4 × 10^11^ cm^−2^, with the gate voltage *V*_g_ = −57 V. **d** Crossover phenomenon by considering classical diffusion (*R*_NL_ ∝ *R*_L_) and valley Hall transport (*R*_NL_ ∝ *R*_L_^3^). The experimental data (solid circles, *V*_g_ = −60 V) clearly show two different regimes which are fitted by two linear curves (orange dashed line with slope 1 and blue dashed line with slope 3). The critical temperature is around 160 K~200 K, as marked by the blue arrow. **e** *R*_NL_ plotted as a function of *V*_g_ at low temperatures. The ohmic contribution, calculated according to *R*_L_ and device geometry, is deducted from the measured *R*_NL_ at different temperatures. **f** 1/*R*_NL_ (orange circles) and 1/*R*_L_ (blue circles) in log scale plotted as functions of 1/*T* at *V*_g_ = −60 V. Three distinct transport regimes were observed: the thermal activation (TA) transport, nearest neighbor hopping (NNH) transport, and the variable range hopping (VRH) transport. **g** Semilog plot of *R*_NL_ as a function of L at *n* = 2 × 10^11^ cm^−2^ (orange squares). Nonlocal signal decays exponentially with increasing *L*. The dashed line yields a valley diffusion length of ∼1 μm

The temperature dependence of *R*_L_ and *R*_NL_ uncovers the mesoscopic mechanism of both the local and nonlocal transport. The conduction can be separated into three regimes: the thermal activation (TA) at 250 K > T > 130 K, the nearest-neighbor hopping (NNH) at 130 K > T > 60 K, and the variable-range hopping (VRH) below 60 K (sample A of *L* = 3.6 μm and *W* = 1.5 μm, see Fig. 2f and Supplementary Fig. 7a and 7b). These transport regimes are consistent with previous studies^37,38^. Since pronounced nonlocal signals are observed in all three transport regimes, there appears no clear connection between the transport regimes and the onset of strong nonlocal signals. Interestingly, the characteristic temperatures of both NNH and VRH for *R*_NL_ are much larger than those for *R*_L_ in the range of *V*_g_ ∼ −60 to −58 V (Supplementary Fig. 7d and 7e). This indicates a higher energy barrier in the nonlocal transport and an anomalous origin of the nonlocal signal.

To determine the origin of the observed *R*_NL_, we investigate the scaling relation between *R*_NL_ and *R*_L_ as functions of *V*_g_ at different temperatures for both sample A (Fig. 2) and sample B (Supplementary Fig. 8). For a fixed *V*_g_, both *R*_L_ and *R*_NL_ increase when the temperature is lowered. In sample A, two regimes with distinct scaling behaviors become clearly visible in Fig. 2c, d, the logarithmic plot of *R*_L_ and *R*_NL_ at different *V*_g_. Above 160 K, the slopes of the ln*R*_NL_ versus ln*R*_L_ curves are 1, indicating that *R*_NL_ ∝ *R*_L_. Below 160 K, the slopes turn to 3 in the low electron density regime (*R*_L_ ≈ 10^8^ to 10^9^ Ω), which amounts to $R_{{\mathrm{NL}}} \propto R_{\mathrm{L}}^3$. Indeed, a diffusive model has predicted such power-law relations^36^, in which a cubic scaling holds for a spin or valley Hall effect^36^. As introduced above and calculated later, the massive Dirac band structure of monolayer MoS_2_ produces large valley Hall conductivity $\sigma _{{\mathrm{xy}}}^{\mathrm{V}}$ (see below) but much weaker spin Hall conductivity^36^ (see Supplementary Note 1), Therefore, it is natural to attribute the observed nonlocal signal to the VHE, and the obtained cubic scaling may be analyzed by the predicted formula^36^

1 $$R_{{\mathrm{NL}}} = \frac{1}{2}\left( {\frac{{\sigma _{{\mathrm{xy}}}^{\mathrm{V}}}}{{\sigma _{{\mathrm{xx}}}}}} \right)^{{\hskip -2.5pt}2}\frac{W}{{\sigma _{{\mathrm{xx}}}l_{\mathrm{V}}}}{\mathrm{e}}^{ - \frac{L}{{l_{\mathrm{V}}}}} \propto \left( {\sigma _{{\mathrm{xy}}}^{\mathrm{V}}} \right)^{\hskip -2pt 2}R_{\mathrm{L}}^3.$$

where *l*_V_ is the valley diffusion length (or inter-valley scattering length), and σ_XX_ and *R*_L_ have the simple relation of $\sigma _{{\mathrm{xx}}} = \frac{L}{{R_{\mathrm{L}}W}}$. We will focus on such a VHE-based hypothesis now and elaborate more on the exclude of spin Hall effect in Discussion.

The *R*_NL_ and *R*_L_ data measured at different temperatures for the case of *V*_g_ = −60 V are plotted in Fig. 2d. The cubic law is not applicable above 160 K, due to the enhancement of inter-valley scattering by the smear of the lowest conduction sub-band spin splitting (estimated as *E*_s_*/k*_B_ ∼ 169 K, see Supplementary Fig. 9) at high temperatures. Below 160 K, Eq. (1) can be employed to estimate *l*_V_. For the case of intermediate inter-valley scattering and edge roughness, *l*_V_ ∼ 0.36 μm if we assume $\sigma _{{\mathrm{xy}}}^{\mathrm{V}}\sim 1e^2/h$. In the limit of strong inter-valley scattering and edge roughness, *l*_V_ ∼ 0.43 μm if we assume $\sigma _{{\mathrm{xy}}}^{\mathrm{V}}\sim 0.1e^2/h$. These values of *l*_V_ are comparable to those obtained in graphene systems^10–14,17,18^.

We further investigated the length dependence of the nonlocal valley transport. Apart from sample A (*L* = 3.6 μm) and sample B (*L* = 6 μm), two more samples (*L* = 11 μm and 16 μm) are investigated (Supplementary Fig. 8). The semilog plot of *R*_NL_ at *n* = 4 × 10^11^ cm^−2^ (extracted from the Hall measurement, see Supplementary Fig. 10) versus the sample length yields an estimate of *l*_V_ ∼ 1 μm (Fig. 2g). This value is very close to *W* and much larger than the electron mean-free path *l*_m_ ∼ 20 nm (estimated from the sample mobility *μ* for the range of *n* where the cubic scaling appears) and the localization length *ξ* ∼ 50 nm (see Supplementary Fig. 11). Nevertheless, these estimates based on the observed nonlocal signals are suggestive of *l*_v_ in the order of micron. In sample B, the cubic scaling remains even at room temperature, attributed to the dominant valence-band contribution to $\sigma _{{\mathrm{xy}}}^{\mathrm{V}}$ and particularly the large intrinsic bandgap that is impossible for graphene systems.

### Nonlocal transport in bilayer and trilayer MoS_2_

**Fig. 3:**
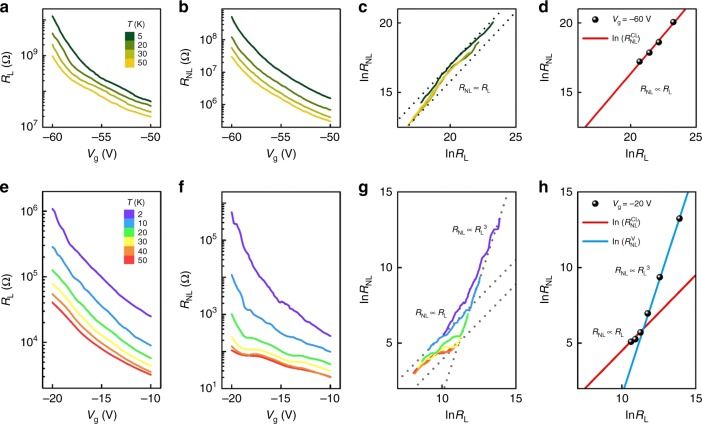
Local and nonlocal resistances of bilayer and trilayer MoS_2_. **a**, **b**, **e**, **f** Gate-dependence of *R*_L_ and *R*_NL_ at different temperatures in bilayer **a**, **b** and trilayer **e**, **f** samples. **c**, **g** Scaling relation between ln *R*_L_ and ln *R*_NL_ is obtained at different temperatures in bilayer **c** and trilayer **g** samples. For the trilayer case, *R*_NL_ scales linearly with *R*_L_ in the high electron density regime, whereas the cubic scaling law *R*_NL_ ∝ *R*_L_^3^ is observed in the low electron density regime (*n*c = 4 × 10^11^ cm^−2^ or *V*_g_ ^=^ −18.4 V). **d** ln*R*_L_ v.s. ln*R*_NL_ for bilayer MoS_2_. In the full range of gate voltages, *R*_NL_ scales linearly with *R*_L_, and the experimental data (black dots, *V*_g_ = −60 V) is fitted by a linear curve (red solid line). **h** ln*R*_L_ v.s. ln*R*_NL_ for trilayer MoS_2_. The experimental data (black dots, *V*_g_ = −20 V) clearly show two different regimes which are fitted by two linear curves (red solid line with slope 1 and blue solid line with slope 3). Evidently, a crossover exists from linear (*R*_NL_ ∝ *R*_L_) to cubic scaling behaviors (*R*_NL_ ∝ *R*_L_^3^)

For bilayer MoS_2_, the measured *R*_L_ and *R*_NL_ as functions of *V*_g_ at different temperatures are plotted in Fig. 3a, b. As the carrier density increases, *R*_L_ and *R*_NL_ decrease in a similar fashion in the temperature range of 5–50 K. This yields a linear scaling behavior between *R*_L_ and *R*_NL_, as analyzed in Fig. 3c, d, and no cubic scaling is detected. We note that extrinsic *P* symmetry breaking can be introduced into atomically thin bilayers via external gating, as achieved in bilayer graphene^11,12^, and that detecting a nonlocal signal in gated bilayer graphene requires a threshold gating strength^11,12^. In our devices, however, *V*_g_ is too low to reach the threshold estimated by an recent optical experiment^18^, the estimated potential difference between the top and bottom layers is ∼ 9.2 meV at *V*_g_ = −60 V. This weak symmetry breaking produces little change in the total Berry curvature as compared with the pristine case (Supplementary Fig. 12), given the facts that the induced potential is much smaller than the bandgap and that the valence-band contribution to $\sigma _{{\mathrm{xy}}}^{\mathrm{V}}$ is dominant. In light of this analysis, the gating-induced *P* symmetry breaking is negligible in our bilayer MoS_2_. Therefore, we conclude that the absence of cubic scaling in bilayer MoS_2_ indicates the crucial role of strong *P* symmetry breaking in generating VHE. This is consistent with the theoretical understanding of VHE^5–9^, as aforementioned in Introduction.

This key conclusion can be immediately tested in thicker MoS_2_ samples. Given that *P* symmetry is broken (respected) in pristine odd-layer (even-layer) MoS_2_, one might wonder whether the intrinsic VHE and its cubic scaling could be detected in trilayer MoS_2_. Figure 3e, f display our *R*_*L*_ and *R*_*NL*_ data measured in trilayer MoS_2_ as functions of *V*_g_ at different temperatures. Evidently, the measured *R*_NL_ rapidly decreases as *V*_g_ increases in the narrow range of −20 V < *V*_g_ < −18.4 V, which is reminiscent of the behavior of *R*_NL_ in our monolayer devices in the low density regime. Similar to the monolayer case, the logarithmic plots of *R*_L_ and *R*_NL_ in Fig. 3g exhibit clear changes in slop from 1 to 3 near *V*_g_ = −18.4 V, further confirming the observation of the nonlocal signal of VHE in trilayer MoS_2_. To illustrate the temperature dependence, Fig. 3h plots the scaling relation between *R*_L_ and *R*_NL_ at different temperatures for the case of *V*_g_ = −20 V. Again, there is a clear change in slop from 1 to 3 near 30 K. Moreover, the valley diffusion length can be extracted based on Fig. 3h and Eq. (1). We obtain *l*_V_ ∼ 0.5 μm and ∼1 μm, respectively, for the aforementioned two limits $\sigma _{{\mathrm{xy}}}^{\mathrm{V}}\sim 1e^2/h\;{\mathrm{and}}\sim \hskip -2pt 0.1\;e^2/h$. Both the observed amplitude of nonlocal signal and the estimated valley diffusion length in the trilayer MoS_2_ devices are comparable to those in the monolayer case. In addition to the crucial role of *P* symmetry breaking, significantly, these observations are suggestive of a universal physical origin of VHEs in odd-layer TMDCs, as discussed below.

### Layer-dependent Berry curvatures

**Fig. 4:**
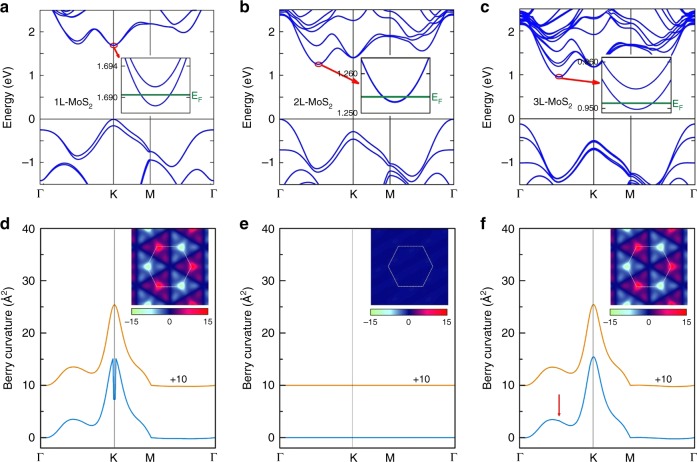
Band structures and Berry curvatures of atomically thin MoS_2_. **a**–**c** Band structure of (**a**) monolayer, (**b**) bilayer, and (**c**) trilayer MoS_2_. The conduction band edges lie at the K-valleys in the monolayer but at the Q-valleys in the bilayer and trilayer. Insets of **a**–**c**: The Fermi levels only cross the lowest sub-bands, which are spin degenerate in **b** but spin split in **a** and **c**. **d**–**f** Berry curvatures of **d** monolayer, **e** bilayer, and **f** trilayer MoS_2_. The blue curves are the total curvatures of all occupied states below the Fermi levels (~2 meV from the conduction band bottom), whereas the orange curves are the total curvatures of all valence-band states. The red arrow in **f** points out a tiny bump at a Q-valley. Insets of **d**–**f** 2D mapping of Berry curvatures in the 2D Brillouin zone (white dashed lines)

To better understand the thickness dependent observations, we calculate the electronic band structures and Berry curvatures^15,16^ for monolayer, bilayer, and trilayer MoS_2_. The band structures in Fig. 4a, c are indeed thickness dependent. In particular, the conduction-band minima lie at the K-valleys for the monolayer, whereas they shift to the Q-valleys for the bilayer and trilayer. Given the low electron densities in our samples (∼4 × 10^11^ cm^−2^ in monolayers and trilayers, ∼1 × 10^12^ cm^−2^ in bilayers), the Fermi levels only cross the lowest conduction sub-bands, as indicated by the green lines in Fig. 4a, c. As bilayer MoS_2_ has a restored *P* symmetry that is intrinsically broken in odd-layer MoS_2_, the sub-bands are spin degenerate in the bilayer yet spin split in the monolayer and trilayer. With these band structures, we further compute the Berry curvatures that drive the VHEs. Berry curvature vanishes if both *P* and *T* are present. As plotted in Fig. 4d, f, our calculations reveal that the curvatures are indeed trivial in the bilayer yet substantial in the monolayer and trilayer. This explains the reason why no cubic scaling is observed in bilayer MoS_2_ and highlights the role of *P* symmetry breaking in producing VHEs.

It is puzzling to understand and compare the nonlocal signals of VHEs in monolayer and trilayer MoS_2_. Similar cubic scaling behaviors and their transitions to linear ones above the critical densities or temperatures are observed in both cases. However, the conduction-band Berry curvatures (the difference between the blue and orange curves in Fig. 4d, f) are large in the monolayer K-valleys yet negligibly small in the trilayer Q-valleys. This implies that the geometric explanation of VHE requiring finite doping^5^ should not be the origin^11^, which is further evidenced by the fact that the cubic scaling behaviors weaken rapidly with increasing the electron densities.

On the other hand, these facts appear to be in harmony with the topological VHE^6–9^ that arises from the valley Hall conductivity (see Supplementary Note 1). This conductivity amounts to the total valley-contrasting Berry curvature contributed from all occupied states, i.e., all the states below the Fermi level if at zero temperature. In our case, the monolayer and trilayer share almost identical substantial valence-band Berry curvatures (the orange curves in Fig. 4d, f), due to the extremely weak interlayer couplings. By contrast, the conduction-band contributions are different but very minor (the difference between the blue and orange curves in Fig. 4d, f) because of the low electron densities. Therefore, the valence-band contributions dominate the valley Hall conductivities, leading to similar nonlocal signals of VHEs in monolayer and trilayer. Recently, nearly quantized edge transports have been observed along the designed or selected domain walls in graphene systems^13,14^ and even in artificial crystals^19^. In our case, the roughness of natural edges can cause edge inter-valley scattering^8^ and remove any possible edge state^39^. This also partly reduces the valence-band contributions^4^ which in principle would result in a quantized valley Hall conductivity (valley Chern number^6,8^) in the massive Dirac model.

## Discussion

Finally, we note that the VHE and spin Hall effects are distinct in TMDCs, in spite of the spin-valley locking. The spin-valley locking is a property at Fermi level only when it lies in the lowest conduction or highest valence sub-band. Yet, all states below Fermi level contribute to the spin and valley Hall conductivities^6^ (see Supplementary Note 1). Although a similar line of analysis based on Eq. (1) can be done for a theoretical hypothesis of spin Hall effect as well, it appears that this is not the case for three reasons. First, the spin Hall conductivities are predicted to be very small for pristine odd-layer TMDCs when the valence bands are fully filled^16^ (see Supplementary Note 1). Second, the observed nonlocal resistances have little response to a magnetic field up to 9 T (Supplementary Fig. 13). Third, the spin diffusion length in TMDCs is at the scale of several tens of nanometers^40,41^, which is 1–2 orders smaller than the extracted diffusion lengths based on our experimental data or Eq. (1).

In conclusion, the pronounced nonlocal signals are observed in our MoS_2_ samples with length up to 16 μm and at temperature up to 300 K. The valley diffusion lengths are also estimated to be in the order of micron. The low carrier concentration ensures the low possibility of bulk inter-valley scattering and maintains a long valley diffusion length. In addition, the mirror and *T* symmetries lock the spin and valley indices of the lowest sub-bands, preventing bulk inter-valley scattering via spin conservation. Our observed intrinsic VHEs and their long valley diffusion lengths are promising for realizing room-temperature low-dissipation valleytronics. To better elucidate the outstanding problems of both geometric^5^ and topological^6–9^ VHEs, our observations and analyses call for future efforts, particularly complementary experiments in *p*-type TMDCs (where spin Hall conductivities are predicted to be much larger^16^) such as the one^42^ that we became aware of during the peer review process.

## Methods

### Van der Waals structures

MoS_2_ bulk crystals are bought from 2D semiconductors (website: http://www.2dsemiconductors.com/), and the h-BN sources (grade A1) are bought from HQ graphene (website: http://www.hqgraphene.com/). To fabricate van der Waals heterostructures, a selected MoS_2_ sample is picked from the SiO_2_/Si substrate by a thin h-BN flake (5–15 nm thick) on PMMA (950 A7, 500 nm) via van der Waals interactions. The h-BN/MoS_2_ flake is then transferred onto a fresh thick h-BN flake lying on another SiO_2_/Si substrate, to form a BN-MoS_2_-BN heterostructure (step 1 in Supplementary Fig. 1).

### Layer numbers and stacking orders

To determine the number of layers for a MoS_2_ sample, we carried out micro-Raman and photoluminescence measurements before making a device (Supplementary Fig. 14). We also took cross-sectional STEM (JEOL JEM-ARM200F Cs-corrected TEM, operating at 60 kV) images after the electronic measurement. The STEM image can clearly determine the number of MoS_2_ layers (Supplementary Fig. 14) and distinguishes the 2H stacking order from other stacking orders such as 1T and 3R (Supplementary Fig. 15).

### Selective etching process

A hard mask is patterned on the heterostructure by the standard e-beam lithography technique using PMMA (step 2 in Supplementary Fig. 1). The exposed top BN layer and MoS_2_ are then etched via reactive ion etching (RIE), forming a Hall bar geometry (steps 3 & 4 in Supplementary Fig. 1). Then a second-round e-beam lithography and RIE is carried out to expose the MoS_2_ layer (steps 5 & 6 in Supplementary Fig. 1). The electrodes are then patterned by a third-round e-beam lithography followed by a standard e-beam evaporation (steps 7 & 8 in Supplementary Fig. 1). To access the conduction band edges of MoS_2_, we choose Titanium as the contact metal, as the work function of Titanium (∼4.3 eV) matches the band-edge energy of MoS_2_ (∼4.0–4.4 eV depending on the layer numbers).

### Electronic measurement

The *I*–*V* curves are measured by Keithley 6430. Other transport measurements are carried out by using: (i) low-frequency lock-in technique (SR 830 with SR550 as the preamplifier and DS 360 as the function generator, or (ii) Keithley 6430 source meter (>10^16^ Ω input resistance on voltage measurements). The cryogenic system provides stable temperatures ranging from 1.4 to 300 K. A detailed discussion of the nonlocal measurement is presented in Supplementary Fig. 6.

## Supplementary information

Supplementary Information

## Data Availability

The authors declare that the major data supporting the findings of this study are available within the paper and its Supplementary Information. Extra data are available from the authors upon reasonable request.
