# Supplementary material for: Intrinsic valley Hall transport in atomically thin MoS2
Source: Nat Commun. 2019 Feb 5;10:611. doi: 10.1038/s41467-019-08629-9 (PMC6363770; doi:10.1038/s41467-019-08629-9)
Supplement: Supplementary file 1 — Supplementary Information [file 41467_2019_8629_MOESM1_ESM.pdf]

Supplementary Information

Intrinsic valley Hall transport in atomically thin MoS_2

Wu et al.

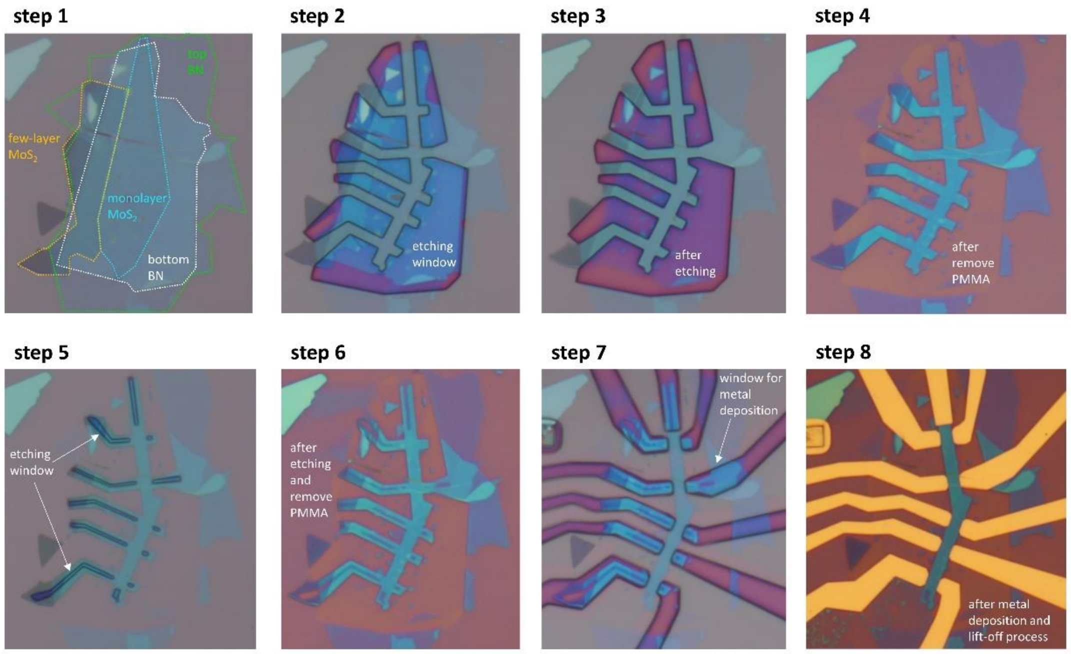

Supplementary Figure 1 | Device fabrication.

Process flow of the device fabrication:

Step 1: Making the BN/MoS_2/BN heterostructure by the dry transfer method; then perform thermal annealing at 350 °C for 6 hours.

Step 2: Define an etching window by electron beam patterning.

Step 3: Use reactive ion etching to fabricate the Hall bar structure.

Step 4: Remove PMMA and do thermal annealing at 350 °C for 4 hours.

Step 5: Define the etching window for the second time by electron beam patterning.

Step 6: Use reactive ion etching to expose the edge of MoS_2 layer.

Step 7: Define the window for metal deposition.

Step 8: Deposit Ti/Au (5/80 nm) and lift-off the PMMA/metal film.

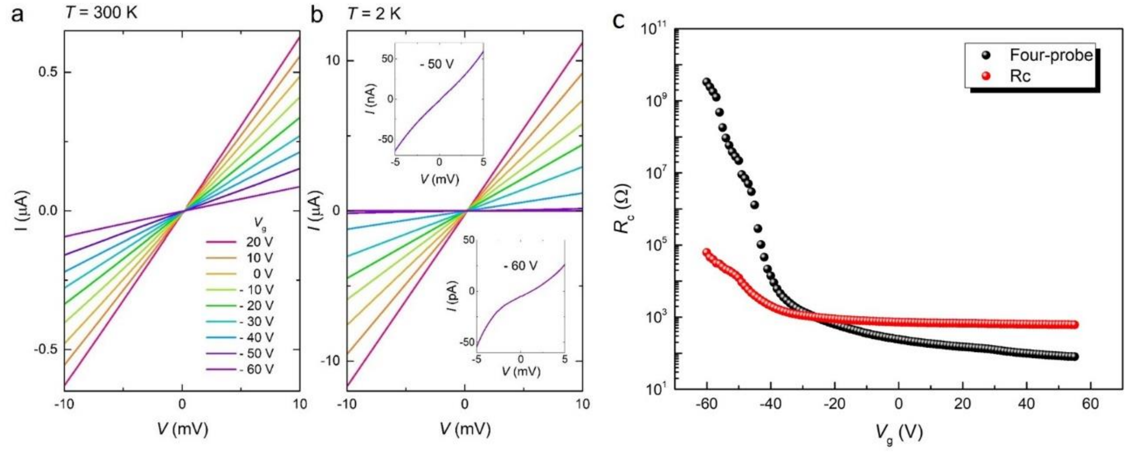

Supplementary Figure 2 | *I-V* curves of monolayer MoS_2.

(**a**,**b**) The *I-V* curves for monolayer MoS_2 at various gate voltages at 300 K (**a**) and 2 K (**b**). The two inset graphs in (b) show the zoom in features at $V_g = -50$ and -60 V at 2K, respectively. The nonlinear shape indicates the emergence of the hopping transport. (**c**) The contact resistance R_c (red curve) ranges 10^3~10^5 Ω depending on the back gate voltages. At high carrier density, the contact resistance saturates to ~ 1 k Ω ·μm. At low carrier density ($V_g = -57$ V), the contact resistance is at a very low level compared with the resistance of MoS_2 channel materials.

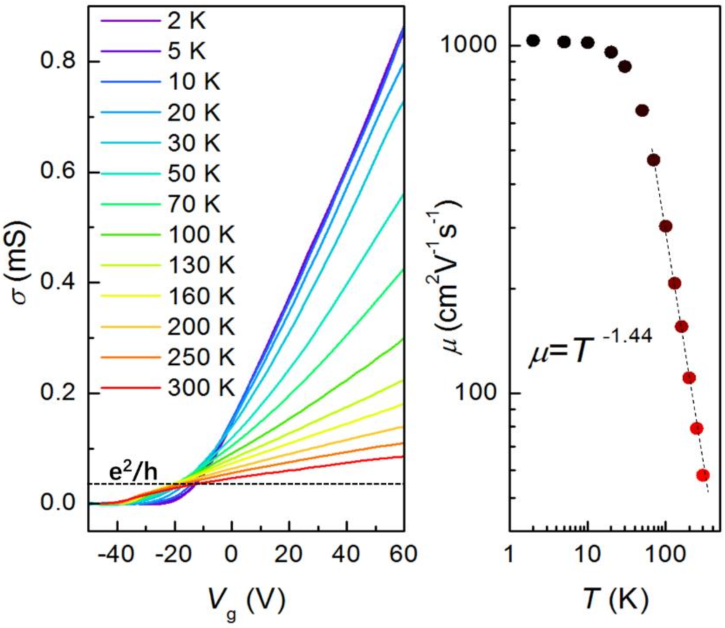

Supplementary Figure 3 | Temperature dependent output behaviors of monolayer MoS_2 (sample A).

Conductance σ vs. gate voltage V_g curves at different temperatures show a clear metal-insulator transition (MIT) point. The MIT point locates at $n_M \sim 5.5 \times 10^{12}$ cm^{-2} (V_g = −10 V). The temperature dependence of mobility demonstrates that phonon is the main reason for mobility drop at high temperatures. At 2 K, the mobility reaches ~1,000 $cm^2V^{-1}s^{-1}$.

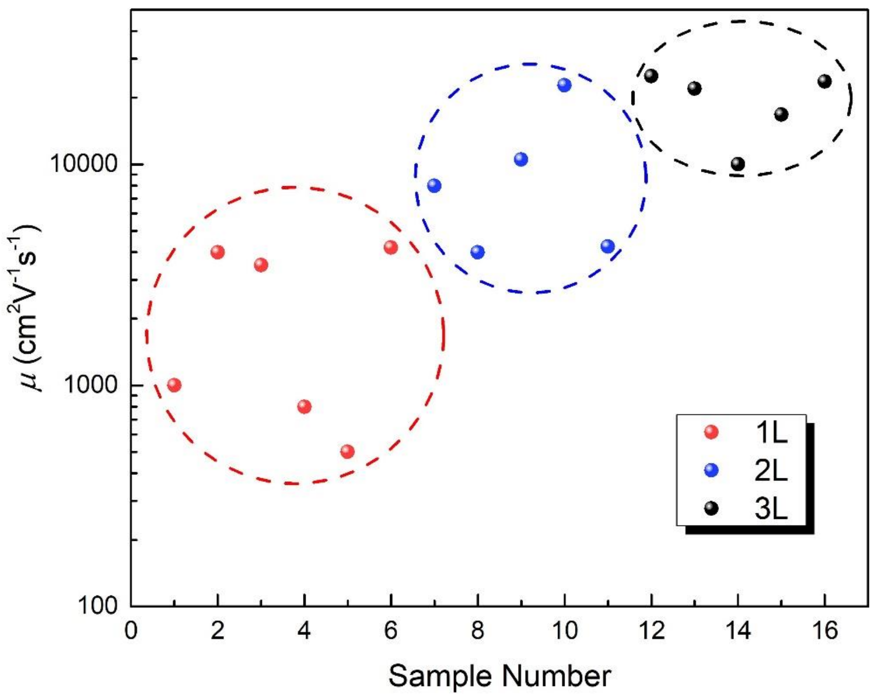

Supplementary Figure 4 | Layer-dependent mobilities.

The field-effect mobility ranges 500-4000 cm^{2}V^{-1}s^{-1} for monolayer (1L) MoS_2 samples, 4000-23000 cm^{2}V^{-1}s^{-1} for bilayer (2L) ones, and 10000-25000 cm^{2}V^{-1}s^{-1} for trilayer (3L) ones at 2 K. Possible reasons for the relatively lower mobilities in 1L and 2L may be the work function mismatch between MoS_2 and the contact metal, relatively higher defect densities, and weaker screening effect in thinner samples.

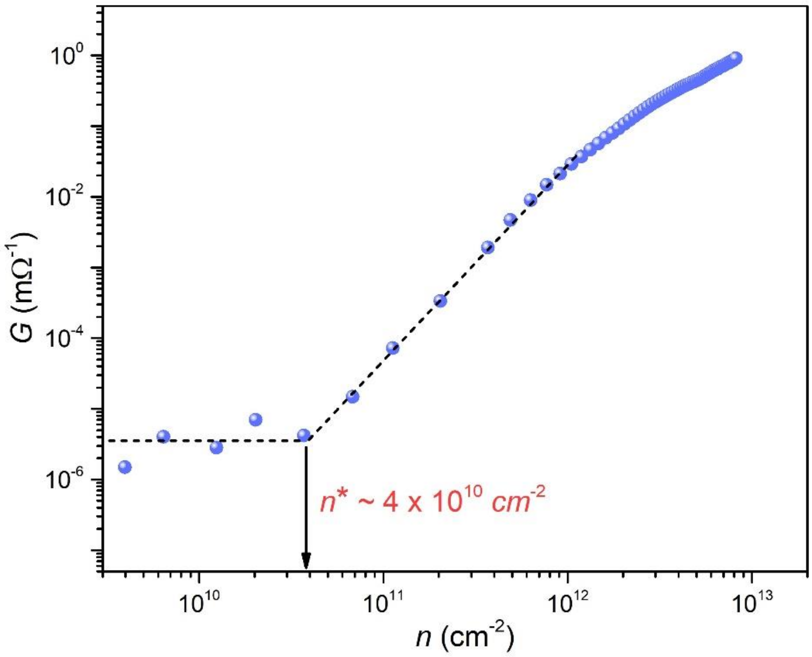

Supplementary Figure 5 | Estimation of residue carrier density in monolayer MoS_2. The residue carrier density n^* can be estimated as follows. Conductance G as a function of carrier density n is plotted in a double-logarithmic scale. This plot shows a low level of charge inhomogeneity, $n^* = 4\times10^{10}$ cm^{-2} (pointed to by the arrow). This value is one order higher than that for high-quality graphene samples[1,2], mainly due to high defect density in MoS_2.

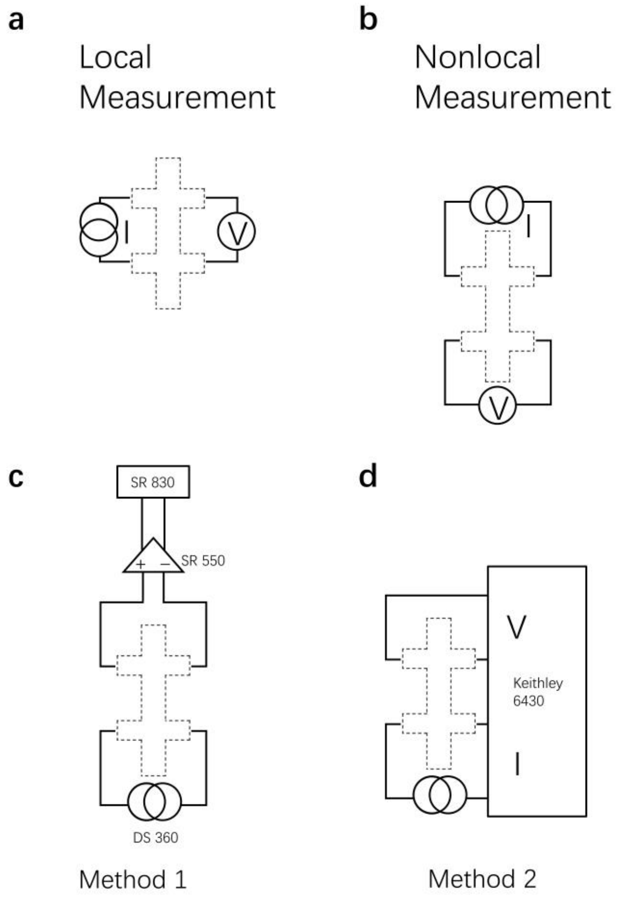

Supplementary Figure 6 | Electronic measurement setup.

(**a**,**b**) A comparison between local (**a**) and non-local (**b**) measurement is shown. (**c**,**d**) The transport measurements are carried out by using: (**c**) low-frequency lock-in SR 830 (10 MΩ input impedance) with SR550 (100 MΩ input impedance) as the preamplifier and DS 360 as the function generator when the resistance is below 10^7 Ω, and (**d**) Keithley 6430 source meter (> 10^{16} Ω input resistance on voltage measurements) when the resistance is above 10^7 Ω. The signal detection method is similar to those discussed in two previous papers[3,4].

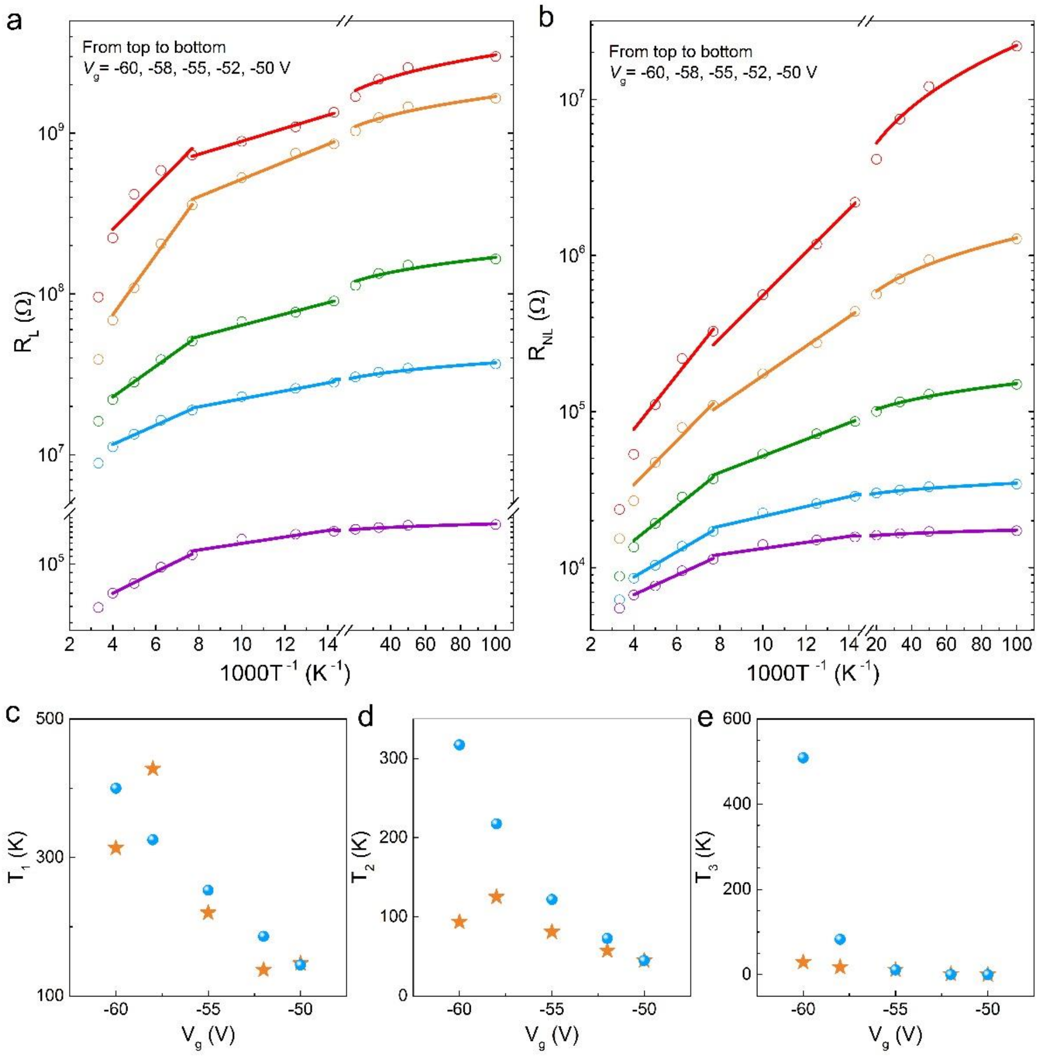

Supplementary Figure 7 | Thermally activated and hoping transport in monolayer MoS_2.

(**a,b**) $R_{\mathrm{L,NL}}$ as functions of 1000/T at various gate voltages. Below T = 250 K, the conduction can be separated into three temperature regimes. (i) Thermally activated transport. In this case the number of carriers that can be thermally activated to delocalized states reduces with decreasing the temperature. The conduction follows the Arrhenius form $1/R_1^{\mathrm{L,NL}}\exp\left(-T_1^{\mathrm{L,NL}}/T\right)$. Here $k_B T_1$ is equivalent to the potential barrier that separates the charge puddles. (ii) Nearest-neighbor hopping (NNH) transport. As the temperature decreases, the conduction occurs by thermally assisted hopping between adjacent localized states, which is the so-called NNH transport. The

conduction also follows the Arrhenius form $1/R_2^{\mathrm{L,NL}}\exp\left(-T_2^{\mathrm{L,NL}}/T\right)$. (iii) Variable-range hopping transport. When the temperature further decreases, the variable-range hopping transport dominants. Considering both the distance and energy separations between localized states, an average nearest-neighbor "distance" determines the overall conductivity. The conduction follows $1/R_3^{\mathrm{L,NL}}\exp\left(-(T_3^{\mathrm{L,NL}}/T)^{1/3}\right)$ in such systems.

Above all, the temperature dependence of maximum R_L and R_{NL} can be modeled by the formula below consisting of three terms:

$$\frac{1}{R_{\mathrm{L,NL}}}=\frac{1}{R_1^{\mathrm{L,NL}}}\exp\left(-\frac{T_1^{\mathrm{L,NL}}}{T}\right)+\frac{1}{R_2^{\mathrm{L,NL}}}\exp\left(-\frac{T_2^{\mathrm{L,NL}}}{T}\right)+\frac{1}{R_3^{\mathrm{L,NL}}}\exp\left(-(\frac{T_3^{\mathrm{L,NL}}}{T})^{\frac{1}{3}}\right),(1)$$

where $R_1^{\mathrm{L,NL}}$, $R_2^{\mathrm{L,NL}}$and $R_3^{\mathrm{L,NL}}$ are fitted proportionality factors. $k_{\mathrm{B}}T_1^{\mathrm{L,NL}}$, $k_{\mathrm{B}}T_2^{\mathrm{L,NL}}$, and $k_{\mathrm{B}}T_3^{\mathrm{L,NL}}$ can be considered as the potential barriers of the thermal activation transport, nearest neighbor hopping transport, and the variable range hopping transport, respectively, where k_{B} is the Boltzmann constant. (**c-e**) The characteristic temperatures T_1 (**c**), T_2 (**d**), and T_3 (**e**) for R_L and R_{NL} are extracted, respectively. There is no much difference between T_1^{L} and T_1^{NL}, so as $T_{2,3}^{\mathrm{L}}$ and $T_{2,3}^{\mathrm{NL}}$ out of the cubic scaling range. However, when $V_g\sim -58$ to -60 V, i.e., in the cubic scaling range, $T_{2,3}^{\mathrm{NL}}$ is several times larger than $T_{2,3}^{\mathrm{L}}$, revealing a high hopping barrier in the nonlocal transport and implying an anomalous origin of the nonlocal signal.

Here, we note that there is no clear relationship between the transport regime and the onset of the strong non-local signal. Our analysis of the temperature dependent resistances is to mention that the nonlocal transport is not in the metallic region, i.e., the carrier density is below the MIT point.

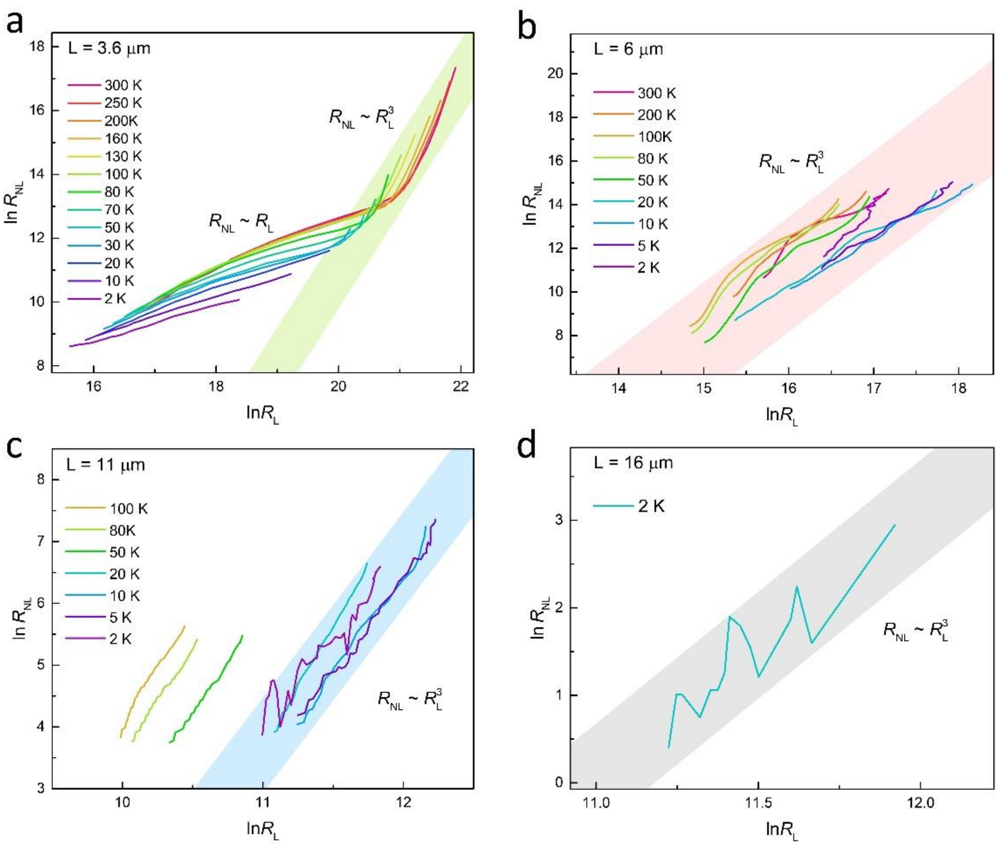

Supplementary Figure 8 | Scaling relation between R_L and R_{NL} in monolayer MoS_2 at several lengths.

(**a**) Replot of Fig. 2c for comparison. (**b-d**) Scaling relation for samples with the lengths of 6 μm (**b**), 11 μm (**c**), and 16 μm (**d**). For the 6 μm long sample, the cubic scaling maintains even at room temperature. Such cubic scaling remains at 100 K for the 11 μm sample. For the 16 μm long sample, the cubic scaling disappears above 2 K.

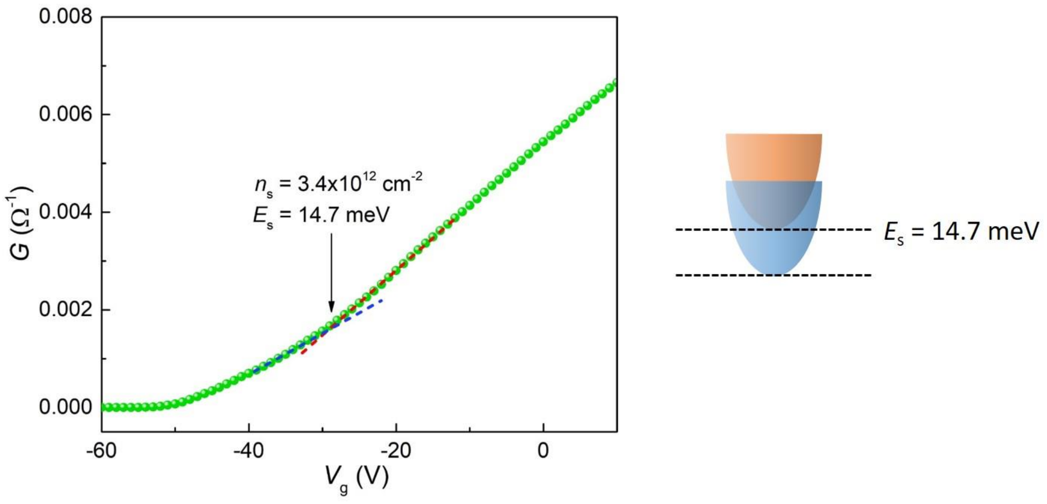

Supplementary Figure 9 | Estimation of the SOC induced spin splitting.

First-principles calculations predict that the SOC induced spin splitting at conduction band minima in monolayer MoS_2 (at K/K' points) is ~3 meV. However, recent experiments reported that this value can be several times larger than the predicted value, ranging from 6.2~15 meV[5-7]. Here, we estimate this spin splitting E_s by considering the slop change point in the conductance G vs V_g plot. The slop change point ($V_g = -29$ V) yields a carrier density $n_s = 3.4\times10^{12}$ cm^{-2} and $E_s = 14.7$ meV. Such a large spin splitting value, together with the spin-valley locking near conduction band minima, can largely reduce the inter-valley scattering possibility (a spin flip is needed in addition to the momentum change). Thus, the VHE signal can maintain at high temperatures. For our monolayer MoS_2, the VHE disappears at 160 K < T < 200 K (Fig. 2d), which is in agreement with the extracted spin splitting value $E_s/k_B \sim 169$ K.

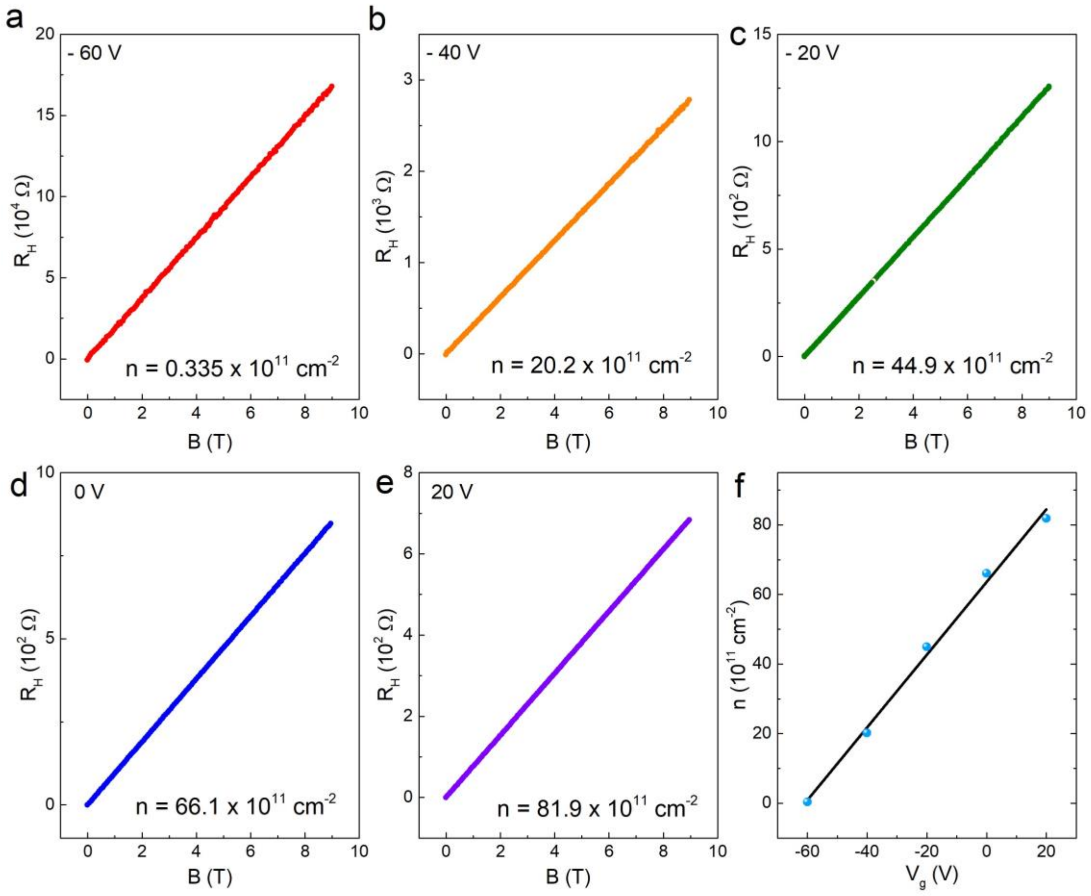

Supplementary Figure 10 | Carrier densities in monolayer MoS_2.

(**a-e**) Hall resistances at various gate voltages for monolayer MoS_2 (sample A). The relevant electron densities are calculated based on the linear fits. (**f**) Carrier density as a function of the gate voltage (blue dots). The linear fit (dark line) is in excellent agreement with the gate capacitance model $n = \alpha(V_g - V_0)$, yielding $\alpha = 1.04 \times 10^{11}\ \mathrm{V^{-1}cm^{-2}}$ and the threshold voltage $V_0 = -60.9$ V. At T = 2 K and V_g = - 57 V (where the valley Hall signal start to become pronounced), the threshold carrier density is $n_{\mathrm{VHE}} = 4.06 \times 10^{11}\ \mathrm{V^{-1}cm^{-2}}$. Based on the band structure obtained from our density-functional-theory calculations, the Fermi energy is estimated as $E_{\mathrm{F}} = 1.8$ meV, given the constant density of states ($n_{\mathrm{th}} = \frac{m^*}{\pi\hbar^2} E_{\mathrm{F}}$). Note that this is an upper limit of the Fermi energy, without considering any localized states in the gap.

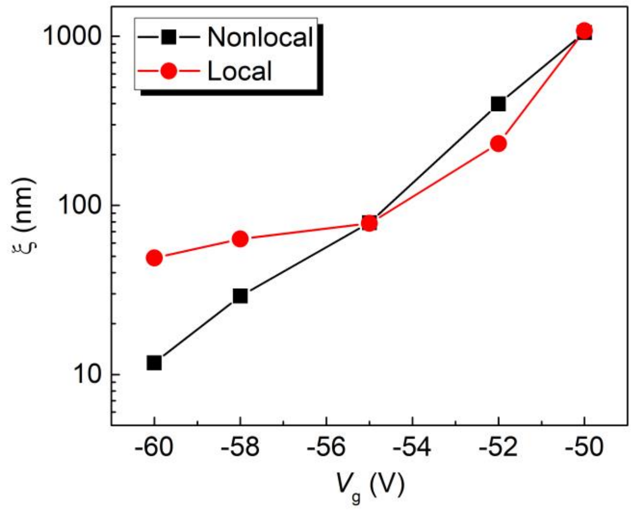

Supplementary Figure 11 | Localization lengths in monolayer MoS_2.

The variable-range hopping localization length ξ can be extracted from the formula: $\xi = \sqrt{\frac{13.8}{k_B \rho_D T_3}}$, where k_B is the Boltzmann constant, $\rho_D = \frac{m^*}{\pi\hbar^2}$ is the constant density of states when the Fermi level only crosses the lowest sub-band, T_3 is the characteristic temperature of the variable-range hopping mechanism[8]. The effective mass $m^* = 0.55\ m_e$ is taken from the temperature-dependent Shubnikov-de Haas oscillations in another work of our group[7]. The extracted localization length (~several tens of nm in the VHE region) is about two orders smaller than the valley diffusion length (~1 micron).

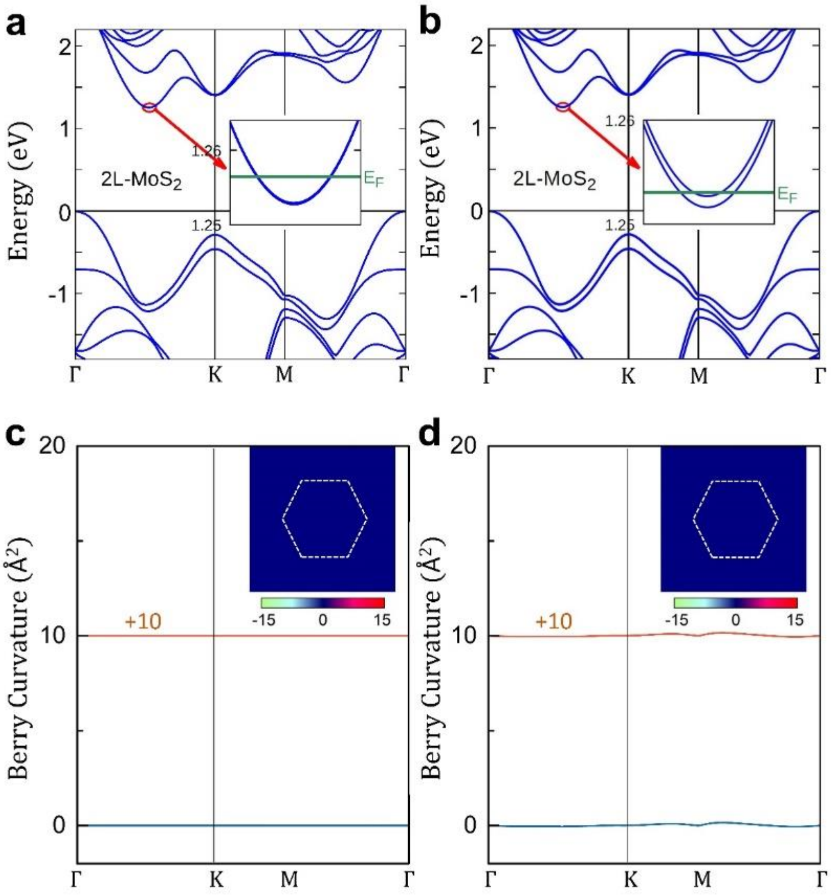

Supplementary Figure 12 | Gate induced symmetry breaking in bilayer MoS_2.

(**a,b**) The band structure of bilayer MoS_2 (**a**) without and (**b**) with a layer potential difference. (**c,d**) Berry curvatures of bilayer MoS_2 (**c**) without and (**d**) with the layer potential difference. The blue curves are the total curvatures of all occupied states below the Fermi levels, whereas the orange curves are the total curvatures of all valence-band states. Insets of (**c,d**) 2D mapping of Berry curvatures in the 2D Brillouin zone (white dashed lines). The potential difference U is calculated using the formula $U = \frac{n_1 - n_2}{C_Q} = \frac{C_g}{2C_{MS} - C_Q} V_g \approx -\frac{C_g}{C_Q} V_g$ (equation 12 in the supplementary information of ref. 14). Here, n_1 and n_2 are the charge carrier densities in the top and bottom layer, respectively, $C_g = 1.53 \times 10^{-8}$ Fcm^{-2} is the gate capacitance, $C_{MS} \approx 6 \times 10^{-6}$ Fcm^{-2} is the interlayer geometric capacitance, and $C_q = \frac{2m^* e^2}{\pi\hbar^2} \approx 10^{-4}$ Fcm^{-2} is the quantum capacitance. At $V_g = -60$ V, the calculated U is ~ 9.2 meV. The band structure (**b**) and the Berry curvatures (**d**) are calculated upon this assumption.

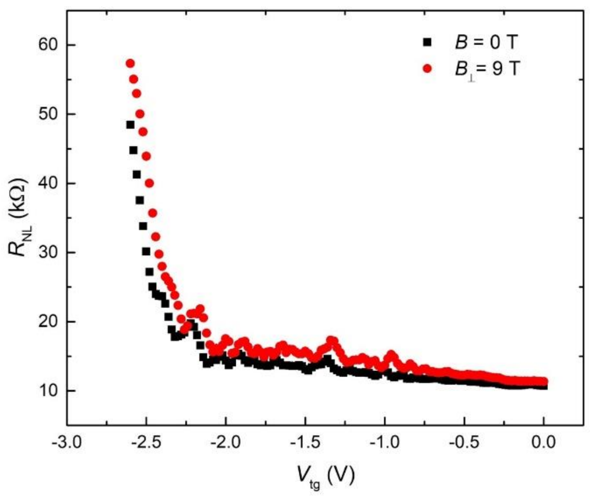

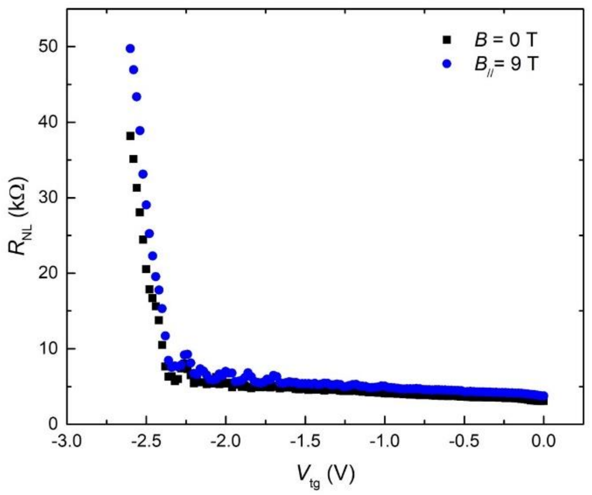

Supplementary Figure 13 | Magnetic field response of the nonlocal resistances.

The left/right figure shows the nonlocal resistances as a function of gate voltages under perpendicular/in-plane magnetic fields. The nonlocal resistances show little response to the magnetic field.

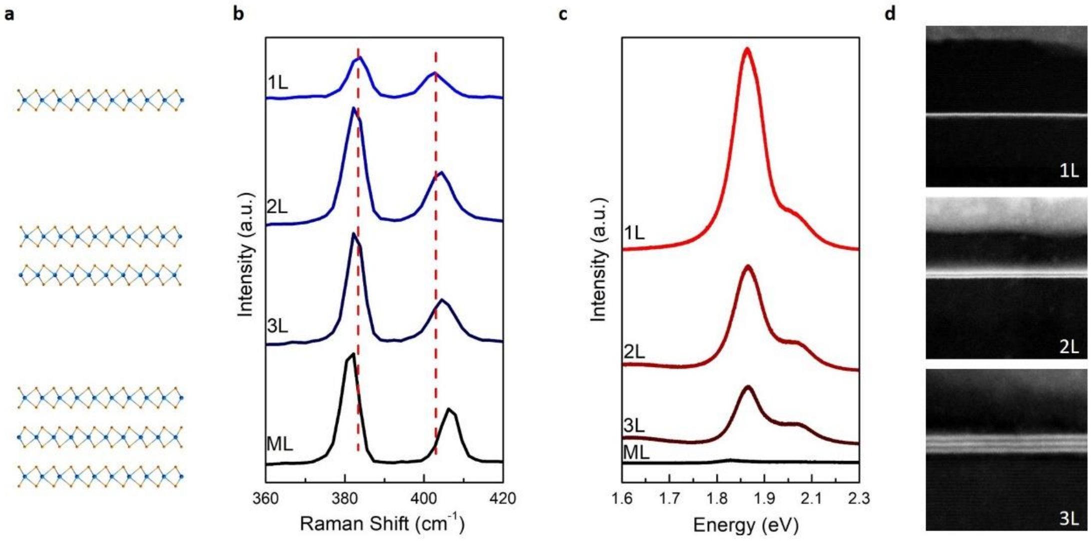

Supplementary Figure 14 | Determination of layer numbers for MoS_2 samples.

(**a**-**c**) To determine the layer numbers for mono-, bi-, and tri-layer 2H-MoS_2 (the stacking structures are shown in (**a**)), we carried out micro-Raman (**b**) and photoluminescence (**c**) measurements before making devices. (**d**) We also took cross-sectional STEM images after electronic measurements. The layer numbers, as well as the stacking type (see Supplementary Fig. 14), can be fully confirmed through the high-resolution STEM technique.

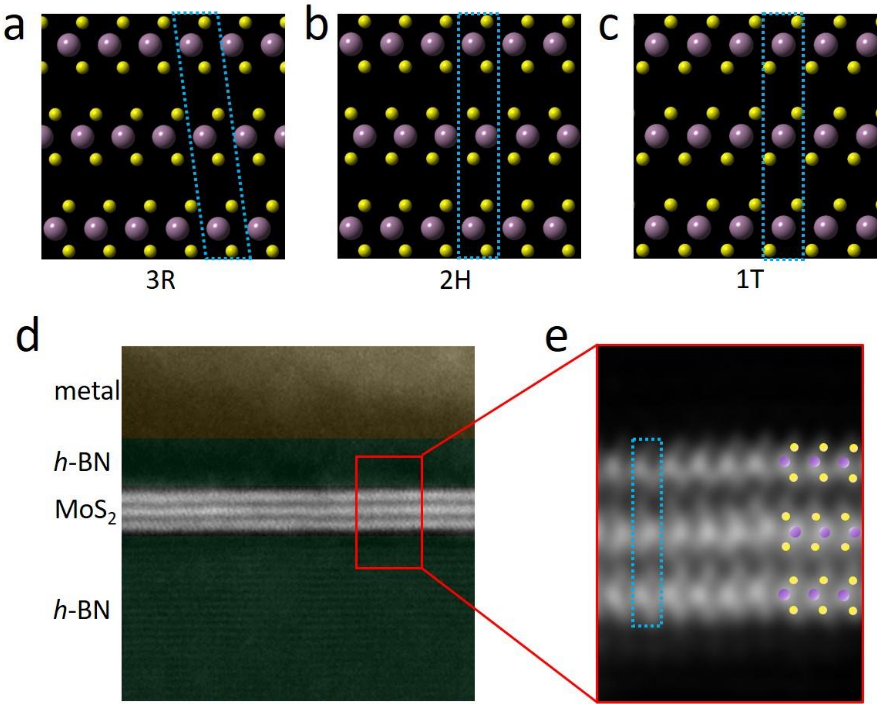

Supplementary Figure 15 | Imaging MoS_2 crystal structures.

(**a**-**c**) The side views of 3R (**a**), 2H (**b**), and 1T (**c**) stacking orders along [100] direction. For the 3R phase each layer has the same S-Mo-S bond orientation, as shown in (**a**). For the 2H phase each layer is rotated by 180° with respect to the adjacent layers, as shown in (**b**). For the 1T phase, the nearest sulfur atoms to each molybdenum atom are at the top-right and down-left sides, forming a straight angle along the S-Mo-S direction, as shown in (**c**). To distinguish whether the crystal structure is 3R, 2H, or 1T for our channel material, the cross-sectional STEM was carried out (JEOL JEM-ARM200F Cs-corrected STEM, operating at 60 kV). (**d**) The STEM image of our trilayer MoS_2 FET device, encapsulated by top and bottom *h*-BN layers. (**e**) The zoomed in image clearly identified the 180°-rotated S-Mo-S bond orientation structure, demonstrating that our MoS_2 is of 2H phase.

Supplementary Note 1 | Spin Hall and valley Hall effects

Both the valley-Hall and spin-Hall effects can produce nonlocal transport. However, spin-valley locking does not indicate the equivalence between spin and valley Hall effects. Basically, spin-valley locking is a property at Fermi level only when it lies in the lowest conduction sub-band or the highest valence sub-band. Yet, all states below Fermi level (including the occupied conduction-band states, all the valence-band states, and the in-gap impurity states) contribute to the spin and valley Hall conductivities.

Theoretically, spin (*s*) and valley (*v*) Hall conductivities have distinct definitions: $\sigma_v = (\sigma_{up,K} + \sigma_{down,K}) - (\sigma_{up,K'} + \sigma_{down,K'})$ and $\sigma_s = (\sigma_{up,K} - \sigma_{down,K}) + (\sigma_{up,K'} - \sigma_{down,K'})$. Experimentally, the spin (valley) Hall effect addresses the difference in Hall conductivity between the two spin (valley) species. Consider a TMD monolayer, which has a broken inversion symmetry. (a) The time-reversal symmetry ensures that $\sigma_{up,K} = -\sigma_{down,K'}$ and $\sigma_{up,K'} = -\sigma_{down,K}$. (b) Our Fig. 4 shows that the Fermi level is very close to the conduction band bottom, so the Hall conductivities are dominated by the valley-band contribution. (c) In each valley, the spin-up and spin-down electrons can be described by the same massive Dirac fermion model but with different band gaps[9,10]: $M_{up,K} = M_{down,K'} = \Delta + \Delta_{SOC}$ and $M_{down,K} = M_{up,K'} = \Delta - \Delta_{SOC}$. Because of $\Delta >> \Delta_{SOC}$ the difference in band gap can be ignored such that $\sigma_{up,K} \approx \sigma_{down,K}$ and $\sigma_{up,K'} \approx \sigma_{down,K'}$. Based on the facts (a)-(c), we obtain $\sigma_v \approx 4\sigma_{up,K} \sim e^2/h$ and $\sigma_s \approx 0$; the latter is also shown in Fig. 4a of ref. 11. (Note that the disorder states can reduce σ_v and σ_s.) The case for a TMD trilayer is similar. For a TMD bilayer, the restored inversion symmetry requires $\sigma_v = \sigma_s = 0$ precisely.

Therefore, the spin-Hall and valley-Hall effects can be distinguished in TMD odd-layers, and σ_v is way larger than the vanishingly small σ_s. Namely, the pronounced nonlocal signatures in our experiments arise from the valley-Hall effect.

Supplementary References

1 Grushina, A. L. et al. Insulating state in tetralayers reveals an even–odd interaction effect in multilayer graphene. Nat. Commun. **6**, 6419 (2015).

2 Feldman, B. E., Martin, J. & Yacoby, A. Broken-symmetry states and divergent resistance in suspended bilayer graphene. Nat. Phys. **5**, 889 (2009).

3 Shimazaki, Y. et al. Generation and detection of pure valley current by electrically induced Berry curvature in bilayer graphene. Nat. Phys. **11**, 1032-1036 (2015).

4 Sui, M. et al. Gate-tunable topological valley transport in bilayer graphene. Nat. Phys. **11**, 1027-1031 (2015).

5 Lu, J. et al. Evidence for two-dimensional Ising superconductivity in gated MoS_2. Science **350**, 1353-1357 (2015).

6 Pisoni, R. et al. Interactions and Magnetotransport through Spin-Valley Coupled Landau Levels in Monolayer MoS_2. Phys. Rev. Lett. **121** (2018).

7 Lin, J. et al. Probing Landau levels of strongly interacting massive Dirac electrons in layer-polarized MoS_2. Preprint at https://arxiv.org/abs/1803.08007 (2018).

8 Moser, J. et al. Magnetotransport in disordered graphene exposed to ozone: From weak to strong localization. Phys. Rev. B **81**, 205445 (2010).

9 Xiao, D., Liu, G., Feng, W., Xu, X. & Yao, W. Coupled spin and valley physics in monolayers of MoS_2 and other group-VI dichalcogenides. Phys. Rev. Lett. **108**, 196802 (2012).

10 Li, X., Zhang, F. & Niu, Q. Unconventional quantum Hall effect and tunable spin Hall effect in Dirac materials: Application to an isolated MoS_2 trilayer. Phys. Rev. Lett. **110**, 066803 (2013).

11 Feng, W. et al. Intrinsic spin Hall effect in monolayers of group-VI dichalcogenides: A first-principles study. Phys. Rev. B **86**, 165108 (2012).
